# Adaption and implementation of the engage programme within the early childhood curriculum

**DOI:** 10.1038/s41598-022-25655-8

**Published:** 2022-12-14

**Authors:** Dione Healey, Barry Milne, Matthew Healey

**Affiliations:** 1 Department of Psychology, University of Otago, Box 56, Dunedin, 9054 New Zealand; 2 Centre of Methods and Policy Application in the Social Sciences, University of Auckland, Auckland, New Zealand; 3 Independant Consultant: Research Methods and Data Analyses, Dunedin, New Zealand

**Keywords:** Developmental biology, Psychology

## Abstract

Poor self-regulation has been associated with an array of adverse outcomes including difficulties with school transition, educational attainment, and social functioning in childhood, and employment, mental health, physical health, relationships, and criminal activity in adulthood. Enhancing Neurobehavioural Gains with the Aid of Games and Exercises (ENGAGE) is a play-based intervention fostering the development of self-regulation in pre-schoolers and has led to improvements within the home setting. The aim for this study was to ascertain whether ENGAGE can be implemented within an Early Childhood Education (ECE) group setting and whether this leads to improved self-regulation. This trial has been registered with the Australian New Zealand Clinical Trials Registry (ANZCTR); trial number ACTRN12622000364774; trial web address: https://www.anzctr.org.au/ACTRN12622000364774.aspx. 668 children aged 3–5 years and their teachers, across 28 ECEs participated. Children’s self-regulation skills were assessed via scores on the Hyperactivity, Aggression, and Attention Problems subscales of BASC-2. Results indicted no significant changes in self-regulation skills across a 10-week waitlist period. Following 10 weeks of the ENGAGE programme, significant improvements in self-regulation were reported, and these were maintained at 2- and 6-month follow-up. These findings indicate that ENGAGE translates well into the ECE setting and has the potential to have population-based impacts which could lead to more positive societal outcomes.

## Introduction

The early childhood education (ECE) setting plays a significant role in successfully preparing children for the transition to formal schooling. It is now well known that successful school transitions require strong self‐regulation^1,2^. There are many definitions of self-regulation, but it is broadly defined as the ability to plan and manage behaviour and regulate emotions^3^, which generally refers to the capability of controlling one’s cognitive focus (thoughts), emotions (feelings), and behaviour (actions) in a goal directed manner^4,5^. Without good self-regulation children struggle to focus and engage with learning material at school. This is evidenced by numerous study findings indicating that self‐regulation predicts academic achievement^6–11^. Poor self-regulation in early childhood has also been linked to later unemployment, criminality, poverty, and mental and physical health difficulties^12^. Poor self-regulation in childhood is evidenced by hyperactivity, inattention, and aggression. These attributes are associated with childhood disorders such as Attention Deficit Hyperactivity Disorder and Conduct Disorder. However, it is clear that improvements in self-regulation, even in those with average or high levels of self-regulation, are associated with improvments in later life outcomes^12^.

Early childhood education (ECE) settings are an opportune environment for teaching self-regulation as many young children are enrolled in at least some form of early childhood care, providing the opportunity for population-based approaches. ECE settings are generally heavily play-based and focused on child-directed learning. Children learn self-regulation through play^13^. For example, many games involve turn-taking, remembering rules, focusing on what others are doing to keep up with the game, planning their next move, and managing frustration when things are not going well in the game. Hence, play is an ideal forum in which to teach/practice self-regulation.

Several studies have examined game-based approaches to skill development within the preschool setting. For example, Tominey and McCleland^14^ have developed a programme focused on improving preschoolers’ executive functioning, a core component of self-regulation. Their programme is called Red Light, Purple Light (RLPL) and involves 6 different circle time games played twice a week over eight weeks, in groups of five to eight children. The games increase in complexity over time and focus on improving attention, working memory, and inhibitory control. Several studies have trialled this intervention and found that it leads to more improvements in the targeted skills (i.e., attention, working memory, and inhibitory control) and academic achievement when compared control conditions involving the early education curriculum as usual^14–16^. Keown et al.^17^ conduced a randomized controlled trial of RLPL with similar results to those of the studies mentioned prior; but this study also measured changes in more general self-regulatory abilities within the classroom to indicate generalisability of skills. Results indicated no statistical differences between the RLPL and control groups in generalisable self-regulatory skills, as measured by teacher ratings on the Child Behaviour Rating Scale. It is possible that the interventions narrow focus on six games and three core skills limits generalisability.

Other school-based approaches to self-regualton training have led to similar findings. Two well extablished preschool programmes are the Promoting Alternative Thinking Strategies (PATHS)^18^ curriculum which teaches children how to use self-talk and problem solving as ways to improve inhibitory control; and the Tools of the Mind^19^ which is an integrated comprehensive programme where teachers are taught how to implement and support activities fostering executive control throughout the preschool day. Results indicated that both of these interventions led to improvements in inhibitory control; and that Tools of the Mind also led to improvements in working memory, and cognitive flexibility; but not in problem behaviours. Similarly a review of whether the preschool PATHS programme leads to reductions in problem behaviours (i.e., internalising and externailisng scores) found that many PATHS studies did not show reductions in problem behavious, with more robust findings for it leading to improved social competencies^18^. A more recent longitudinal evaluation of the Tools of the Mind curriculum found no positive effects on children’s academic, executive function, self‐regulation, and social gains from prekindergarten to the end of first grade^19^. This pattern of fingings is in line with those from computer-game based interventions for training working memory (e.g., Cogmed^20^) which have led to improvement in the targeted skills but these improvements have not transferred outside of the practiced tasks^21^^.^ A theme within all of these programes is that they focus on improving aspects of executive functioning as a mechanism for improving behaviour, but this does not appear to generalise; possibly in part due to the narrow focus on the cognitive aspects of self-regulation and not including the behvaioural and emotional aspects to the same degree.

Another embedded preschool curriculm-based programme with a wider scope and more direct focus on generalisability, is the Preschool Situational Self-Regulation Toolkit (PRSIST)^22^ which involves extensive teacher training around self-regaultion and adult supportive practices, and the provision of 28 play-based activities focused on extending children’s self-regulatory capacity. The activities are provided within storylines in a series of books, with each story relating to one of the domains of self-regalation (i.e., behavioural, cognitive, and social-emotional). Each activity includes instructions around increasing the difficulty as children become more proficient, and also how the abilities required to do the activity relate to children’s every day self-regulation. Teachers are asked to complete a minimum of three activities a week and are encouraged to select activities of various types and categories. Results of an RCT comparing PSIST to the usual Austrailan preschool curriculm found that it lead to small improvements in chidlren’ executive functioning abilities but no significant improvements in any of the self-regaultion and academic learning measures obtained^22^. Hence despite it’s focus on broader self-regulation domains and on generalisability it appears that this programme also leads to changes in cognitive functioning that do not translate to behavioural change.

With a similar focus on a more wholistic approach to self-regulation training (including emotional, cognitive and behavioural regulation), and improving generalisability of treatment effects as was used in to PRSIST, Healey and colleagues^23,24^ developed a play-based intervention aimed at fostering the development of self-regulation in pre-schoolers; namely ENGAGE (Enhancing Neurobehavioural gains with the Aid of Games and Exercise). The programme focuses on structure, consistentency, scaffolding, skill building, and generalisation. It involves (1) identifying areas of self-regulation skill development (e.g., taking turns, persisting, calming down, remembering, perspective taking), (2) selecting games that utilise the target skills, (3) talking to children about the game, the skills it involves, and every day instances in which they use these skills, (4) then systematically playing these games at increasingly challenging levels in order to build the skills; and then (5) once skills have been built through play, children are reminded of them in instances within their every day functioning where they would benefit from using the skills. For example, the Animal Speeds game involves doing various activities at different speeds. Cheetah mode means fast, Girrafe mode means moderate, and Tortoise mode means slow. The children need to change the speeds at which they are doing the activity each time the animal speed is called out. Later when children are, for example being too active, they can be asked to go into Giraffe mode as they did in the Animal Speeds game. Any games can be used within the ENGAGE programme—the focus is on teaching skills through play, not on being required to play any specific games. ENGAGE does include an array of examples of games that target specific skill areas^24^, along with examples of every day instances when children use the skills, ways in which to make each game simpler and more complex in order to meet the children’s skill areas, and prompts for how to help children to generalise the skills learnt within the games, to their every day functioning. However, the strength of the programme is that it was specifically designed so that no specialist equipment is required and that it can be adapted to suit a multitude of contexts and cultural approaches. ENGAGE is a framework within which games can be chosen by those administering it; and then the ENGAGE skill building and generalisation approach can be applied to those games. This provides both autonomy and flexibility where the focus is on an approach to teaching skills rather than having to follow a regimented programme; which is unique to ENGAGE.

ENGAGE has been successful in improving self-regulatory abilities of hyperactive pre-schoolers when administered in a home-based setting. Here, parents played games with their children for half an hour a day over an 8-week period; and were encouraged to link the game skills to their children’s everyday functioning at any time that the skills were required. An initial open trial of the intervention showed that regular, targeted, incremental game-play lead to significant reductions in hyperactivity (behavioural regulation), aggression (emotional regulation) and attention problems (cognitive regulation) following the 8-week intervention; as well as improvments in working memory and visuomotor precision^23^; which is indicative of both cognitive changes as well as genralised bahvioural improments. A randomised controlled trial comparing ENGAGE to the well-known parenting behavioural management training programme, Triple P (Positive Parenting Programme) also indicated that it led to significant reductions in hyperactivity, aggression and attention problems as rated by parents and teachers. There were no significant differences in treatment effects for ENGAGE and Triple P indicating that while they address self-regulation skill development very differently, both result in similar improvements in self-regulation. The study also found that there were no significant improvements in self-regulation during an 8-week waitlist period, suggesting improvements were the result of the interventions^24^.

Given its effectiveness within the home setting, particularly in relation to behaviour change, and the fact that the ECE environment is likely to capture the largest sample of the population, the aim of this study was to explore whether ENGAGE could be successfully implemented in a group setting with all children within an ECE, and whether it would lead to improvements in self-regulatory abilities in these children. If effective in this setting, the programme would then have the potential to be used both within home and school settings which is the ideal way to build children’s capacity.

## Methods

### Experimental design

This was an open trial and thus all participating ECE centres received the ENGAGE intervention. Data was collected in five phases: T1. An initial baseline assessment which was followed by a 10-week waitlist period of “education as usual” at each centre; T2. Post-waitlist assessment; T3. Post-ENGAGE assessment, which occurred after 10 weeks of active ENGAGE implementation within the ECE centre; T4. Re-assessment 2 months post-ENGAGE implementation; and T5. Re-assessment 6 months post-ENGAGE implementation.

### Participants

ENGAGE was administered within 28 Early Childhood Education (ECE) centres in Auckland, New Zealand. Across the centres there were a total of 940 children aged 3–4 years. Parental consent was given to collect data on 668 of these children. The only eligibility criteria for child participation were that they were enrolled at a participating ECE centre and that they were aged between 3–4 years.

ECEs were recruited within the Auckland region of New Zealand. This is the largest and most diverse region in the country and therefore enabled an ethnically and economically diverse group of centres to be recruited. Centres were contacted via already established connections with the Auckland Kindergarten Association (AKA). Centres who had had prior exposure to the ENGAGE programme, or who were actively involved in other learning support interventions were precluded from participating. All of the centres who were invited to participate agreed to take part in the research.

#### Enhancing neurobehavioural gains with the aid of games and exercises (ENGAGE)

This intervention involved teachers spending a minimum of 30 min a day exposing children to ENGAGE activities within a group format. Games were typically played at set times (often during morning or afternoon mat times). Some games were played within larger groups (e.g., 15 children) and some in smaller groups (e.g., 3–5 children), depending on the types of games being played. The games were typically run by 1 or 2 teachers at a time. Teachers were instructed to think about skill areas that they wanted to work on (e.g., memory, waiting turn, persisting) and then to select suitable games and apply the ENGAGE approach (explained above) to each game.

Shortly before beginning the 10-week intervention period of the study, teachers attended training workshops run by accredited ENGAGE trainers who were also trained ECE teachers and support workers. Teachers were also provided with a programme manual outlining the programme philosophy, as well as step by step implementation information and examples of games. Teachers were encouraged to introduce any other games that they knew and that involved the targeted skills (i.e., aspects of emotional, behavioural, and/or cognitive regulation).

Once the intervention began the ECEs received regular check-in visits by the ENGAGE trainers/facilitators throughout the 10-week period, enabling frequent trouble shooting and discussion of any concerns.

#### Education as usual: Te Whariki, the New Zealand early childhood curriculm

All early childhood centres in New Zealand are required to follow the Te Whariki curriculm in order to maintain their license to practice. Te Whariki places a strong emphasis on home-school partnerships and child focsued and led learning. It emcompases 4 principals (Empowerment—children will be empowered to learn and grow; Holistic development—children learn and grow in a holistic way. Their intellectual, social, cultural, physical, emotional and spiritual learning is interwoven across all their experiences; Family, whānau and community—a child’s family, whānau and community are recognised as part of the learning experience; and Relationships—children learn through positive relationships with people, places and things). Te Whāriki’s four principles are interwoven with five learning areas: Mana atua (wellbeing), Mana tangata (contribution), Mana whenua (belonging), Mana reo (communication) and Mana aotūroa (exploration). For a detailed description see the New Zealnd Ministry of Education’s publication: https://www.education.govt.nz/assets/Documents/Early-Childhood/ELS-Te-Whariki-Early-Childhood-Curriculum-ENG-Web.pdf.

## Measures

### Behavioral measures

Behavior Assessment System for Children (BASC-2)^25^ is a well-validated and normed scale designed to assess wide ranging areas of child functioning, as rated by parents and teachers. Of particular interest to this study were the Hyperactivity, Aggression, and Attention Problems subscales of this measure as they are indicative of self-regulatory ability. This measure was selected for the study as it the the same one used in past studies using ENGAGE and therefore allows for direct comparisons.

Teachers were asked to complete these 3 subscales of the BASC-2 at each of the five data collection points within this study. Scores across time points were standardized (M = 50 and SD = 10). The ECE were instructed to have the same teacher complete the ratings for each child, wherever possible. For the ratings at Waitlist (T1) and Baseline (T2), 65.4% of the sample were rated by the same teacher at both timepoints. For Baseline (T2) and Post-ENGAGE (T3), 64.6% of the sample had the same teacher rating them at both time points; 53.5% of the sample had the same teacher rate them at Baseline (T2), Post-ENGAGE (T3) and at re-assessment 2 months post-ENGAGE (T4), and 51.7% of the sample were rated by the same teacher at Baseline (T2), Post-ENGAGE (T3), re-assessment 2 months post-ENGAGE (T4) and re-assessment 2 months post-ENGAGE (T5).

### Procedure

The AKA made face-to-face contact with all participating centres’ teachers to provide them with the broad research aims and programme information. The centres where then grouped into regional areas (Central, West, and South Auckland) and trained in the programme administration by experienced ENGAGE trainers within the AKA team. Once they began implementing the ENGAGE programme within their centres, the AKA team provided 5 h a week of teacher support for the full duration of the 10-week programme.

### Ethical approval

This study received ethical approval from the University of Otago Human Ethics Committee prior to commencement. Informed consent was obtained from both parents and teachers and data was only collected for those children whose parents had provided signed consent. While conducting this study, all ethical standards of the American Psychological Association were adhered to.

### Trial registration

This study was registered with the Australian New Zealand Clinical Trials Registry (ANZCTR); trial number ACTRN12622000364774 (01/03/2022); trial web address: https://www.anzctr.org.au/ACTRN12622000364774.aspx.

### Data analysis

We assessed changes in scores for hyperactivity, aggression and attention problems across time using linear mixed model with random intercepts. For each outcome, models were estimated using data from (i) T1 to T3 for n = 565 children with data across all three measurements; (ii) T1 to T4 for n = 409 children with data across all four measurements; and (iii) T1 to T5 for n = 114 children with data across all five measurements. T1 was the reference category in all models. Because models were restricted to children with data across all time points assessed, controls for time-invariant factors such as gender, age at first assessment, ethnicity, and early childhood centre were not necessary.

## Results

### Behavioural ratings

The statistical analyses are presented in three phases. First, we examined whether there were any differences in self-regulation scores in the waitlist (education as usual) compared to the active ENGAGE phase of the study (see Table 1). The final sample available for these analyses included 565 children for whom we had both parent consent to collect data and completed BASC-2 teacher ratings at all three time points. The reason for the missing data was due to children having left the centre (many of whom turned 5 and went to school as in New Zealand children start school on their 5th birthday) during the data collection phase. Teachers completed ratings on all children with parental consent that were enrolled at the centre at the time of each wave of data collection.

**Table 1:** Differences in BASC scores between the waitlist and active ENGAGE periods.

BASC scores	T1 M (SD) n = 565	T2 M (SD) n = 565	T3 M (SD) n = 565	Pairwise comparisons (z-test)
Hyperactivity	51.72 (10.36)	51.49 (10.27)	48.92 (9.44)	T2 v T1: z = − 0.79, p = 0.429 T3 v T1: z = − 9.54, p < 0.001 T3 v T2: z = − 8.75, p < 0.001
Aggression	51.16 (10.49)	51.06 (10.58)	49.05 (9.36)	T2 v T1: z = − 0.31, p = 0.754 T3 v T1: z = − 6.71, p < 0.001 T3 v T2: z = − 6.40, p < 0.001
Attention problems	51.66 (10.03)	51.81 (9.92)	49.05 (9.36)	T2 v T1: z = 0.51, p = 0.611 T3 v T1: z = − 10.37, p < 0.001 T3 v T2: z = − 10.88, p < 0.001

Results indicated that there were no significant changes in self-regulation scores within the waitlist period (from T1 to T2) where the centres continued with their early childhood curriculum as usual. Following the introduction of the ENGAGE programme, results indicate that there was a significant improvement in self-regulation skills, as indicated by statistically significant reductions in Hyperactivity, Aggression and Attention Problems at T3.

Secondly, we examined whether the treatment gains post-ENGAGE were maintained two months (T4) following the end of the active implementation of the programme (see Table 2). The final sample available for these analyses included 409 children for whom we had both parent consent to collect data and completed BASC-2 teacher ratings at all four time points.

**Table 2:** Differences in BASC scores between the waitlist, active ENGAGE period, and two months following the active ENGAGE period.

BASC scores	T1 M (SD) n = 409	T2 M (SD) n = 409	T3 M (SD) n = 409	T4 M (SD) n = 409	Pairwise comparisons (z-test)
Hyperactivity	50.93 (10.05)	50.88 (10.17)	48.23 (8.66)	47.85 (8.69)	T2vT1: z = − 0.16, p = 0.875 T3vT1: z = − 7.87, p < 0.001 T3vT2: z = − 7.71, p < 0.001 T4vT1: z = − 8.96, p < 0.001 T4vT2: z = − 8.80, p < 0.001
Aggression	50.20 (9.82)	50.22 (10.33)	48.52 (8.73)	48.65 (8.85)	T2vT1: z = 0.05, p = 0.958 T3vT1: z = − 4.69, p < 0.001 T3vT2: z = − 4.74, p < 0.001 T4vT1: z = − 4.32, p < 0.001 T4vT2: z = − 4.37, p < 0.001
Attention problems	51.35 (9.99)	51.52 (9.72)	48.68 (9.43)	47.74 (9.55)	T2vT1: z = 0.47, p = 0.640 T3vT1: z = − 7.59, p < 0.001 T3vT2: z = − 8.06, p < 0.001 T4vT1: z = − 10.25, p < 0.001 T4vT2: z = − 10.72, p < 0.001

Results indicated that for those with four measurement points there were no significant differences in the self-regulation scores across the waitlist period (from T1 to T2) where the ECEs continued with their curriculum as usual. There were significant improvements in self-regulation scores at the end of the 10-week active ENGAGE programme implementation, as evidenced by significant reductions in Hyperactivity, Aggression and Attention Problems scores (T3). These improvements were maintained two months after the active programme implementation had ended (T4).

The final set of analyses examined whether the treatment gains post-ENGAGE continued to be maintained six months (T5) following the end of the active implementation of the programme (see Table 3). The final sample available for these analyses included 114 children for whom we had both parent consent to collect data and completed BASC-2 teacher ratings at all five time points.

**Table 3:** Differences in BASC scores between the waitlist, active ENGAGE period, and both 2 and 6 months following the active ENGAGE period.

BASC scores	T1 M (SD) n = 114	T2 M (SD) n = 114	T3 M (SD) n = 114	T4 M (SD) n = 114	T5 M (SD) n = 114	Pairwise comparison (z-test)
Hyperactivity	49.33 (8.64)	48.94 (9.54)	46.20 (7.44)	46.37 (7.88)	43.14 (6.37)	T2vT1: z = − 0.59, p = 0.556 T3vT1: z = − 4.74, p < 0.001 T3vT2: z = − 4.15, p < 0.001 T4vT1: z = − 4.49, p < 0.001 T4vT2: z = − 3.90, p < 0.001 T5vT1: z = − 4.83, p < 0.001 T5vT2: z = − 4.25, p < 0.001
Aggression	49.35 (9.63)	49.46 (10.91)	47.56 (7.84)	47.59 (7.87)	47.68 (6.68)	T2vT1: z = 0.17, p = 0.866 T3vT1: z = − 2.56, p = 0.010 T3vT2: z = − 2.73, p = 0.006 T4vT1: z = − 2.52, p = 0.012 T4vT2: z = − 2.69 , p = 0.007 T5vT1: z = − 2.40, p = 0.017 T5vT2: z = − 2.56, p = 0.010
Attention problems	49.80 (9.22)	49.58 (9.49)	46.99 (8.90)	46.67 (8.84)	46.93 (7.80)	T2vT1: z = − 0.34, p = 0.730 T3vT1: z = − 4.40, p < 0.001 T3vT2: z = − 4.06, p < 0.001 T4vT1: z = − 4.91, p < 0.001 T4vT2: z = − 4.56, p < 0.001 T5vT1: z = − 4.51, p < 0.001 T5vT2: z = − 4.16, p < 0.001

**Figure 1:**
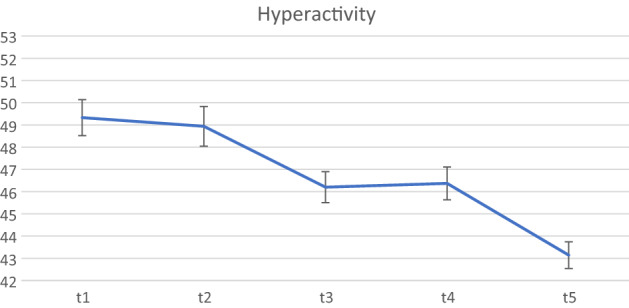
BASC-2 hyperactivity scores from Waitist to 6 month follow-up.

**Figure 2:**
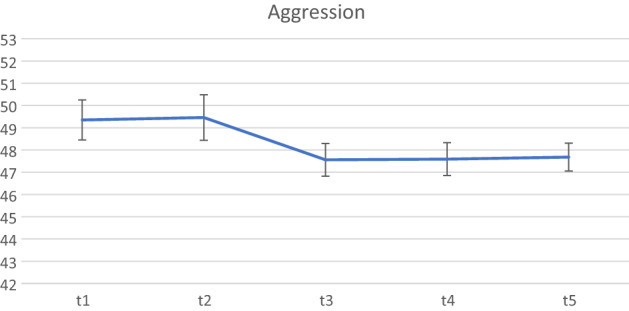
BASC-2 aggression scores from Waitist to 6 month follow-up.

**Figure 3:**
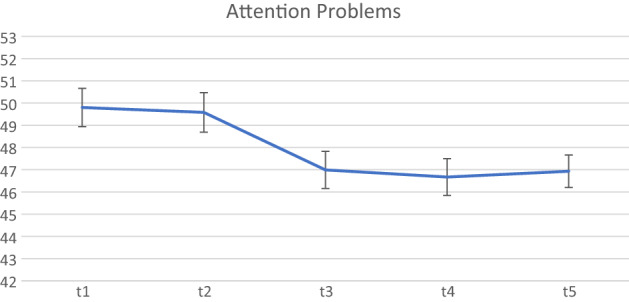
BASC-2 attention Problems scores from Waitist to 6 month follow-up.

Results indicated that again there were no significant differences in the self-regulation scores across the waitlist period (from T1 to T2) where the ECEs continued with their curriculum as usual. There were significant improvements in self-regulation scores at the end of the 10-week active ENGAGE programme implementation, as evidenced by significant reductions in Hyperactivity, Aggression and Attention Problems scores (T3). These improvements were maintained both two (T4) and 6 months (T5) after the active programme implementation had ended. These results are also depicted in Figs. 1, 2, 3.

## Discussion

The aim of this study was to investigate whether the ENGAGE programme, focused on improving self-regulatory skills in pre-schoolers, and originally designed as a programme for parents and their children, would be able to be implemented within a group ECE setting.

ENGAGE translated well to the ECE setting with teachers easily being able to implement it and being very positive about the play-based approach which aligns well with the New Zealand early childhood curriculm.

With regard to changes in self-regulation scores on the BASC-2 ratings by teachers, the Early Childhood Curriculum as usual did not lead to significant changes in self-regulatory abilities over a 10-week period. Once the ENGAGE programme was implemented for 10 weeks, significant improvements in self-regulation were rated by teachers. These improvements were maintained at both 4- and 6-month follow-up. These findings are in line with those of past findings for ENGAGE when implemented with parents and children in the home setting^23,24^; and thus indicate that the programme is similarly effective when implemented by teachers within a group format with the ECE sector.

Numerous early childhood curriculum, play-based programmes exist, most focusing primarlity on executive functioning, a key factor in self-regualation but not all encompassing. All of these programmes been led to improved cognitive function^14–19,22^ but none have led to robust findings of behavior change^17–19^. There are many possible reasons for this, one being the narrow focus on executive functions, another being the lack of purposeful genralisation of skills. Children were found to improve on the core skill areas, but this did not generalise to their every day functioning. The PRSIST programme focused on generalisation by pointing out, within the supplementary materials for each storyline, how the targeted skills were used in daily life; however it is unclear whether teachers actively related the stories and activities to children’s everyday functioning in a purposeful and targeted way as is encouraged within the ENGAGE programme.

Another unique feature of ENGAGE is that teachers are able to select any games to use within the ENGAGE approach to skill building. It is not known whether this autonomy and ability to tailor the programme to each unique setting leads to better genralisability but it does make the programme easier to embed within daily practices. The simple nature of the programme focusing on an approach to teaching skills, rather than having set materials and specific protocols to follow makes it easy to learn and apply without the need for any specialist equipment. Finally, given that the programme appears to be easy to administer, and has been found to be equally effective in the home and early childhood education settings, it can be used across settings to enable strong home-school partnerships and consistency, and maxium exposure to scaffolded skill development using a flexible and fun appraoch.

While these findings suggest that a play-based approach to teaching self-regulation skills is effective and well suited to both the home and the early childhood environment, the current study is not without limitations. The use of a waitlist period prior to administering the programme provided a control for behavioural change, however his study relied solely on teacher report to assess changes in self-regulatory ability. The issues here are two-fold: Firstly, the teachers were involved in administering the intervention and as such may be biased toward reporting improvements; and secondly repeatedly filling in the same rating scale may lead to response biases. Future studies would be well served to include more objective measures such as direct child assessments and observations completed by blinded administrators, parent-report of behaviour change, as well as assessment of a broader range of self-reguation and related constructs such as social functioning, langage abilities, and parenting practices. Future studies should also included complementary focused ENGAGE play activities within the home setting to support the ENGAGE work being done at the ECE centres.

## Conclusion

Despite the limitations discussed above, our results indicate that ECE teachers playing games targeting self-regulation skills in a structured, regular, and incremental manner, that is focused on both skill building and skill generalisation, leads to significant improvements in pre-schoolers’ self-regulation skills; which were not seen when they participated in the ECE curriculum as usual. As such this approach has the potential to have wide population-based effects as we know that poor self-regulation in the preschool years is predictive of a wide array of adverse outcomes across the lifespan. Given that the parent-led version has also been shown to be effective, ENGAGE can easily be administered simultaneously in the home and school setting.

## Data Availability

The datasets analysed during the current study are available from the corresponding author on reasonable request.
